# MULTISECTORAL PARTNERSHIPS TO ADDRESS SUCCESSIVE DISASTER EVENTS IN HOUSTON’S UNDERSERVED COMMUNITIES

**DOI:** 10.1093/geroni/igae098.1608

**Published:** 2024-12-31

**Authors:** Omolola Adepoju, Mary Tipton, Ben Hirsch, Lauren Gilbert

**Affiliations:** University of Houston College of Medicine, Houston, Texas, United States; Humana Integrated Health Systems Sciences Institute, Houston, Texas, United States; West Street Recovery, Houston, Texas, United States; University of Houston, Houston, Texas, United States

## Abstract

**Objective:**

Over the past decade, Houston, Texas, has become one of the most diverse cities in America, during which time it has also endured several federally declared disasters. This study describes the creation of formal academic-community partnerships to build a geospatial-photoethnography dashboard, co-owned and co-developed by the community, to chronicle and assess the impact of successive disaster events on affordable housing and mental health outcomes in three historically medically underserved communities in Houston.

**Methods:**

We used geospatial-longitudinal maps to illustrate housing insecurity and mental health trends from 1980 to 2022, with projections for 2030, 2040, and 2050 at the census tract level. Critical ethnography concepts, such as community conversations and photovoice, are employed to gather and present a storyboard of people’s experiences and coping mechanisms.

**Results:**

Community leaders were identified via prior partnerships with social support groups, healthcare organizations, and faith-based organizations. A total of 15 community partners were invited and 10 agreed to serve as partners on the project. Co-ownership was developed through regular meetings as a large group with all partners, as well as one-on-one meetings with individual groups. To capture the richness of the community conversations, visual notetakers were present at the meetings to provide a graphical representation of the content of the meetings.

**Discussion:**

The community-led initiative amplifies the voices and experiences of those affected by climate change disasters, shedding light on enduring effects on housing and mental health in underserved communities. By embracing community-driven empowerment, the project documents community priorities and proposed solutions.

